# A Comparative Case Study on Active Transport to and From School

**Published:** 2008-03-15

**Authors:** Carrie E Fesperman, Kelly R Evenson, Daniel A Rodríguez, David Salvesen

**Affiliations:** American Planning Association; University of North Carolina at Chapel Hill, Chapel Hill, North Carolina; University of North Carolina at Chapel Hill, Chapel Hill, North Carolina; University of North Carolina at Chapel Hill, Chapel Hill, North Carolina

## Abstract

**Introduction:**

This study investigates active-transport-to-school initiatives through the Active Living by Design Community Action Model framework. The framework outlines five strategies that influence physical activity: preparation, promotion, programs, policies, and physical projects.

**Methods:**

A comparative case study was conducted to investigate active-transport-to-school initiatives at two North Carolina schools. A group of key stakeholders from each site was interviewed (N = 16), including principals, physical education teachers, public safety officers, city planners, regional transportation planners, city council members, and parent representatives. Content analysis was carried out using NVivo software, and data were evaluated using the framework.

**Results:**

Applications designed around all five strategies positively influenced active-transport-to-school programs. Both schools used similar strategies including promotional tactics, policies, and physical projects; however, only one used all five strategies. The scope and duration of these strategies varied by school and ultimately seemed to influence their success. Enablers and challenges to active-transport-to-school programs were identified, including funding, school location, available infrastructure, community involvement, school support, parental buy-in, and sufficient program promotion.

**Conclusion:**

The quality of the strategies, not their mere presence or use, proved important in active-transport-to-school programs. These results suggest that a multidisciplinary approach that develops promotional materials, resources, school support, and environmental changes to sustain factors that influence parental buy-in will prove critical to the success of future walk-to-school initiatives.

## Introduction

In 1969, approximately half of all school-aged children walked or biked to school (also called active transport to school or ATS) (1). In 2001, less than 15% of children and adolescents used ATS (1). In the meantime, the percentage of overweight children increased (2); during 1999 through 2002, 16% of children and teenagers were overweight, up from 5% in 1980 (2). Encouraging children to use ATS could be one way to support healthy behaviors that could reverse these trends, since ATS is associated with higher levels of overall physical activity after school and in the evening (3,4).

The recommendations of the Task Force on Community Preventive Services to increase physical activity in communities (5) included interventions at individual, social, community, environmental, and policy levels. The task force identified effective interventions involving informational approaches (e.g., community-wide campaigns, point-of-decision prompts to encourage use of stairs), behavioral and social approaches (e.g., school-based physical education, individually adapted health behavior change programs), and environmental and policy approaches (e.g., creation of or enhanced access to places for physical activity). ATS was among the effective interventions identified by the task force (6-11).

Many researchers argue that the built environment plays a major role in influencing decisions to walk or bike as a mode of transport to school (7,9). Some of the barriers to ATS that previous studies have identified include distance to school, motor vehicle traffic, crime and danger, restrictive school policies, and neighborhood characteristics such as poor street connectivity, sloped terrain, and lack of sidewalks (6,9,12-16).

Although an increasing number of researchers have called for a multilevel framework to guide ATS interventions (6,10), few models have been developed to direct ATS initiatives. As attention turns toward encouraging ATS in communities, the Active Living by Design (ALbD) (17) Community Action Model could serve as an important guide for researchers, interventionists, community members, schools, and public officials.

The ALbD Community Action Model outlines five strategies, also called the 5 P strategies, that influence physical activity: preparation, promotions, programs, policies, and physical projects (17). These strategies provide direction to interventions that address multiple levels of influence from a socioecological framework (18). *Preparation* is the time deliberately taken to lay the groundwork for an initiative and to strategize ways to reinforce plans for action (8). *Promotions* (i.e., the development of messages and materials through media venues) are an important mode through which to educate and shape public opinion, as well as to gain buy-in from community leaders, key decision makers, and the public (5). *Programs* involve organized activities that either directly or indirectly engage individuals in physical activity in order to garner greater constituent support and provide promotional opportunities (5). *Policies* create a political environment that institutionalizes active living and supports healthy environments (19). Finally, *physical projects* create opportunities for, or remove barriers to, physical activity (5).

Although each of the 5 P strategies is important, we theorized that the combined synergistic effect is greater than the effect of interventions targeting only individuals, environmental changes, or policies (17). This study qualitatively investigates how a comprehensive use of the 5 P strategies might bolster ATS initiatives and improve their chances of success. The findings can provide a more in-depth understanding of how the 5 P strategies work and can inform future research and community initiatives.

## Methods

During fall of 2005, we conducted a comparative case study analysis with representatives of two elementary schools with ATS initiatives and their communities — one school in northeastern North Carolina (School A) and one school in central North Carolina (School B). To respect the privacy of the participants, communities, and schools, we have not identified the schools.

The two ATS initiatives investigated provided an apt comparison because they shared core characteristics yet experienced different levels of success. Site selection was based on having

- multiple public agencies and/or organizations involved in the ATS initiative,
- at least three of the 5 P strategies from the ALbD Community Action Model, including some policy or environmental intervention, as part of the ATS initiative, and
- a heavily used road as a primary access route to school.

### School and community locations and initiatives

Although both ATS initiatives shared core characteristics, the schools were in different communities and served differing student populations (Table 1). School A was in a small town in northeastern North Carolina; School B was in the central area of a larger city in central North Carolina. The locations could affect the success of ATS programs; however, children living in the central area of a city and in small towns have similar odds of using ATS (2.2, city; 2.3, small town) when compared with children living in rural areas (14). Walking and biking rates are also higher among children living in more densely populated neighborhoods (15). The census block group (the smallest geographical unit for which the U.S. Census Bureau publishes sample data) of School B had the higher population density; however, both schools had several residential neighborhoods within a quarter-mile radius.

Both schools had a higher percentage of students eating free or reduced-price lunch than the state average of 48%. Rates of using ATS are estimated to be higher among Hispanic students and students receiving public welfare (14). These rates could suggest that income and transportation options limit transportation choices, and that the absence of transportation options can dictate walking and biking.

The ATS initiatives at each school began in different ways*.* School A was built in 1997 to replace two smaller neighborhood schools. The North Carolina Department of Transportation (DOT) expanded the main access road for School A, already a commuter road at the time, and worked with the city council to construct sidewalks with buffers on both sides of the road. Children began attending School A before road and sidewalk construction was complete, creating congestion around the school entrance. After sidewalks were completed, the school district began enforcing a "no transport zone" bus policy (defining areas where school bus service is not provided) with the hope that students would use ATS. Although some promotion of ATS took place within the community, school administrators had little to no involvement in encouraging children or parents to use ATS.

School B was built in 1961 and already had a large student body that walked to school and an established "no transport zone" despite being on a busy road. When the number of walkers began decreasing, school administrators worked with an organization within the school system, Healthy Kids Healthy Communities, to establish a Walk to School (WTS) Day. That activity was then expanded into a week (WTS Week). The school also received a grant to fund a school-wide program, the Feeling Good Mileage program, which was designed to give students incentives for walking certain distances, including using ATS. A Safe Routes to School workshop, hosted by the city's DOT, resulted in projects that completed nearby sidewalks, cleared objects blocking sidewalks, installed additional safety signals, documented hazardous walking areas in the surrounding area, and relocated the crossing guard to a more visible location. Table 1 outlines each school's use of the 5 P strategies in more detail.

### Interviews and analysis

A multidisciplinary group of professionals (Table 2) was identified as key stakeholders on the basis of their knowledge and involvement in ATS initiatives. After providing informed consent, 16 people were interviewed by the same interviewer using structured interviews; 8 participants were interviewed in connection with School A, 7 in connection with School B, and 1 in connection with both ATS initiatives. Several of the interviewees also were parents or grandparents of children who were attending or had previously attended the elementary schools. Interviews lasted approximately 45 minutes. The interview guide posted on the Physical Activity Policy Research Network's Web site (http://prc.slu.edu/paprn.htm) was tailored to our needs by adding some questions.

Each interview was audiotaped, transcribed, and then coded and checked for consistency by two researchers. Content analysis was completed using NVivo software (QSR International, Doncaster, Victoria, Australia). Initial analysis focused on interview responses to questions on 1) specific initiatives associated with encouraging ATS, 2) ways in which an initiative was implemented, 3) barriers and enablers of initiative implementation, and 4) outcomes of initiatives implemented. We used thematic analysis to further analyze and examine patterns related to the ALbD Community Action Model 5 Ps. Using matrices, we identified patterns based on redundancy (20) and assessed the patterns by site, stakeholders’ professional occupation, and stakeholders’ level of involvement with the ATS initiative. The institutional review board at the University of North Carolina at Chapel Hill, North Carolina, approved this study.

## Results

On the basis of lessons learned and their professional experiences, stakeholders from both communities identified common enabling and hindering factors (Table 3) important to the success of the ATS initiative at each school. Findings are presented for each of the 5 P strategies.

### Preparation

School administrators at both elementary schools stressed that preparations for ATS initiatives (e.g., gathering information and materials, planning promotional messages, soliciting support) must be sufficient so that it is easy for others to support the effort and easy for parents to understand it. For instance, gaining parental buy-in in School A's case was difficult from the outset partly because travel habits were established when no sidewalks existed, thereby hindering use of ATS. Then parents were upset when the school system eliminated the bus service to encourage walking when the sidewalks were finished. Instead of being able to help support or promote an ATS initiative, school administrators handled parental complaints.

In contrast, coordinators of the initiative at School B decided to wait a year before implementing their program so they could be better organized and garner additional teacher support. From the beginning, a coordinated effort took place among school administrators, the school system, and the city's DOT. The school led the effort, while other parties provided support and expertise. By taking an extra year to prepare, the school was able to make it easier for teachers to get involved and support the ATS effort.

### Promotion

Most participants agreed that promoting an ATS initiative to parents and the community is important, but the type, quality, and character of each community's promotional efforts varied widely. At School A, the number of bus routes was reduced in the hope that children would walk to school, and the school system initially promoted the change in policy and the ATS initiative by holding press conferences, getting newspaper coverage, and meeting with parent-teacher associations and school staff. One key informant admitted that "where [the school system] encouraged pedestrians and bike riding, it never really took." Since then, few follow-up promotional efforts have taken place. As congestion increased around the school, additional resources had to be diverted to address traffic concerns.

In contrast, staff at School B and other public officials actively promoted various ATS events. For WTS Day, they coordinated news media coverage for the event, notified parents through an automated calling system, and held weeklong activities for students on various health topics, including pedestrian and bike safety.

#### Parental buy-in

Participants at both schools considered educating and gaining buy-in from parents important to the success of the ATS initiative. One key stakeholder from School A said,

If [parents are] not comfortable with their kid walking, they're not going to let their kid walk or run. There has to be that, some kind of an outreach program I guess, to let the parents know it's been thought through, it's safe, it's connected.

Participants generally acknowledged, particularly participants from School A, that parents were difficult to win over. Interviewees cited multiple reasons for their reluctance, including fear for the child's physical safety, perceived danger of kidnappers, the convenience of dropping their child off, and personal time cost (especially for those who accompanied their children).

#### Source of promotional messages: school administration and teachers

In both communities, key stakeholders working outside of the schools and actively involved with the ATS initiatives (e.g., transportation planners, school system officials) underscored the need for schools and teachers to buy in and promote the initiative. Participants from School A thought that the school administration's languid participation prevented the ATS initiative from reaching its potential. Although School A's administration promoted the initiative early on, neither the current principal nor the previous principal was familiar with efforts to encourage walking to school. A past school administrator from School A recalled,

I think we talked about walking and biking safety to and from school, but as far as [efforts] being done to promote it, that was more of a parental decision, whether or not they wanted their children to be walking on the sidewalks to school or not.

Another stakeholder outside the school stressed that for an ATS initiative to be effective, it had to be not only promoted by the schools but also embraced — "the teachers and the administrators have got to believe in what they're selling."

School B's experiences with promotion echoed the lessons learned by School A. Participants discussed the need for promotion at every level (i.e., principal, administration, and teachers) within the school to generate maximum buy-in. The principal was a key contact for parents and a source of encouragement for other teachers to come on board. Teachers also had contact with parents, but more importantly they worked closely with children, who often had influence with their parents. One participant said,

Our principal has been really supportive of it, and I think when your leader kind of sets the tone, it kind of has trickled down with the staff members. Some community members [became] involved, and the students and teachers [became] excited about it.

### Programs

Although School A did not use programs as a strategy to encourage ATS, School B initiated several programs to support and promote ATS, including WTS Week, WTS Day, and the Feeling Good Mileage program. With the help of Healthy Kids Healthy Communities, WTS Day received coverage in local news media, while the Feeling Good Mileage program created a way to continually highlight the ATS idea for parents and encourage students to use ATS. Interviewees who spoke about School B's program emphasized the importance of designing these programs to win over parents by making it easy for them to participate and by developing a clear message on the program's purpose.

Many public officials connected with School B stressed that the administrators' roles as champions and coordinators within the school were important in implementing the ATS initiative. For instance, a school administrator helped organize a week of activities around WTS Day, applied for the grant to start the Feeling Good Mileage program, and brought together a fitness committee of teachers. Although the school system's Healthy Schools Healthy Communities coordinator served as a contact point for funding and expert support, ultimately the school led the effort.

Many stakeholders connected with the ATS initiative at School B underscored the importance of support from school leadership; school system representatives were especially emphatic. Interviewees who were not closely involved with either ATS initiative, interestingly, did not list school support as an enabling factor.

### Policy influence

The influence of several policies and policy-related factors, including "no transport zones," safety policies, and the commitment of government, particularly through funding, played a role in shaping community members' perceptions of ATS initiatives and affected the implementation of other 5 P strategies.

Both schools had "no transport zone" bus policies. Participants recognized that these zones forced parents to employ other alternatives than busing to get their children to school, whether driving, carpooling, or using ATS, but otherwise the participants did not mention the busing policy as being an enabler for using ATS. The initiation of the "no transport zone" policy at School A created feelings of ill will among parents.

Policies implementing additional safety measures (e.g., police presence, traffic regulation enforcement, crossing guards) did not seem to be significant determinants of an initiative's success. However, those interviewed acknowledged that these measures played a role in reassuring parents that their children were safe using ATS. Representatives from the school system, town council, and police department believed police presence slowed traffic down to create a safer pedestrian environment. Stakeholders valued crossing guards because they maintained traffic control, reassured parents of their child's safety, and made children feel safer when walking. Safety officers and school administrators particularly noted the importance of the crossing guards' role in directing traffic for children to safely cross the street.

Funding is an essential part of state and local governments' commitment to help create a safe environment for ATS through augmentation of infrastructure (near schools and surrounding neighborhoods) and promotion of a school's ATS initiatives (with both promotional materials and resources to pay for staff to handle school or county-wide ATS efforts). Key stakeholders within the school system and the school administration particularly emphasized their limited ability to promote ATS initiatives without funding. Said one, "You know, with any initiative, especially in schools, the first thing — well, how much more work is involved in it? . . . Is it gonna cost the school any more money?" Participants working at all levels — schools, school districts, city, and state — had limited time and money. New initiatives to endorse ATS, without added financial resources, only added to their loads.

### Physical projects

Both communities had completed physical projects to encourage ATS before the interviews. Participants interviewed thought that the presence of sidewalks in particular was an important precursor to a successful ATS initiative. One transportation planner said, "There needs to be sidewalks to walk on because no parent in their right mind is gonna send their kid down a drainage ditch to school." Other infrastructure — including crosswalks, safety signals, bike racks, and trails — were also mentioned as encouraging ATS.

Several stakeholders noted the absence of sidewalks along one approach to School A, where there were several neighborhoods from which children might walk if adequate infrastructure were present. One stakeholder said,

There is an area back over on the other side of [the street], and those children are not a mile and a half away from school, but there's no sidewalk and no way for them to safely cross [the street], so they are picked up by bus.

In contrast, additional sidewalks were built at School B so that sidewalks were on both sides of the street, providing pathways approaching school from either direction. Barriers along the sidewalk, such as an old gate, were also moved to make the walkway more easily navigable for students. Stakeholders underscored the importance of sidewalk availability in neighborhood areas surrounding the schools where children lived.

While other stakeholders mentioned the availability of sidewalks and bike racks, safety officials and city and regional transportation planners expressed the importance of infrastructure most frequently. The groups that remained noticeably silent on the merits of sidewalks were school administrators and teachers.

## Discussion

To understand the success of School B's ATS initiative and the more limited accomplishments of School A, we examined the various efforts each school used through the ALbD Community Action Model framework. Both schools used similar strategies to encourage using ATS, including promotional tactics, policies, and physical projects. However, only School B used preparation and programming, thereby employing all 5 P strategies. The scope and duration of these strategies also varied by school and ultimately seemed to influence the success of School B's ATS initiative. Although both schools used some promotion as a strategy, School B had more modes through which to communicate messages about ATS to parents and students.

The results from the interviews conducted for this analysis illustrate the need for an intervention that draws on strategies addressing several levels of influence and encourages collaboration among various public and school agencies. While ATS coordinators at School A provided the sidewalk infrastructure and political initiative for a "no transport zone" bus policy, they made less effort to garner parental and school support for the initiative. Furthermore, the lack of a program and insufficient preparation to facilitate promotional activities or to directly engage students might have inhibited ATS efforts. At School B, most of the infrastructure was in place, so coordinators could concentrate on promoting ATS initiatives and on implementing programs and other smaller environmental changes to make ATS safer.

The differing fates of these initiatives suggest that the effects of changes in policy or environment diminish if other supports are not in place. Other research has supported this claim, suggesting that the individual, social, and physical environmental determinants of using ATS were of similar importance (6). The next step is to begin to develop a better understanding of ways to plan a multilevel intervention and engage different public entities to better facilitate ATS.

This study tested the applicability of the ALbD Community Action Model (17) to existing ATS initiatives in order to explore its usefulness as an implementation tool for future interventions. Using the framework, we identified some core weaknesses at School A, including lack of preparation and programming as well as weak promotional efforts. The results of the study supported the utility of the ALbD framework, indicating that applications designed around all 5 P strategies positively influence ATS programs. The quality of the 5 P strategies, not their mere presence or use, seemed to be important in achieving successful ATS programs.

To coordinate an intervention using the 5 P strategies, a collaborative effort is needed to bring together professionals with different areas of expertise and domains of influence. Consequently, it is important to explore different models that set the conditions for and guide the implementation of this type of collaboration. School B provides a potential model for collaboration; the school led the initiative and other organizations provided both financial and technical support.

In planning a collaborative effort, coordinators should consider who to involve and how to involve them. Although participants highlighted parental buy-in and involvement as particularly important, neither parents nor the parent-teacher associations were actively involved in either initiative. This could indicate that parental support of an initiative is necessary but parental involvement may not be. The finding that school support was not identified as an enabling factor by people not closely involved with the initiatives could indicate that they assumed the school would be involved, that they may not fully appreciate what could be gained from school involvement, or that they felt other factors were more indicative of a successful program. The fact that teachers did not mention sidewalks as an important factor for ATS programs does not necessarily indicate a belief that sidewalks and other infrastructure are unimportant, but it may suggest that from their perspective other factors seem to be more important determinants of ATS success. 

Of the different factors that influenced use of ATS, the factors that participants mentioned most frequently were school and parental buy-in, infrastructure, adequate funding, and the location of the school (including the congestion and speed of vehicles around it). Some factors identified by the study may apply more to policies affecting future schools than to those affecting existing ones. Factors that cannot be changed easily, such as school location or design, could be key factors for developing future ATS policies and plans for new schools. Other factors, such as garnering school support, addressing congestion around the school entrance, marking crosswalks, and placing crossing guards at busy intersections in the morning and evening are viable goals for ATS interventions at existing schools.

### Limitations

The inability to generalize the study data is a major limitation of this research, although including more than one elementary school provided opportunities to draw inferences and comparisons not allowed by a single-site study. Although we tried to identify schools with similar socioeconomic characteristics and initiative components for this study, the schools studied differed in some ways.

We were also unable to definitively establish whether School B's success was due to the use of all 5 P strategies, the quality in implementing the 5 P strategies, or other factors. The data provide strong evidence that the use of all five strategies — preparation, promotion, programs, policies, and physical projects — contributed to School B's success. Promoting the ATS program was particularly important in achieving use of ATS. Although other factors may also have affected the success of the initiative, this study demonstrates the merit of further investigation into the synergistic effects of employing all 5 P strategies.

Finally, no official, reliable measures of ATS use exist at either school. Success was largely determined by transportation professionals, both at the school district level and at the North Carolina DOT, as well as the coordinators of the initiatives in the schools. Our informal observations and counts of active transport at the schools, however, have corroborated their judgments.

### Conclusion

The ALbD Community Action Model provides a framework for designing an effective intervention. The application of the model in this study suggests intervention efforts are maximized when multiple strategies are employed and indicates a need for further investigation. Promotion emerged as important in facilitating public support and participation as well as being a way to take full advantage of the inroads made through strategies involving the other P's. As effective strategies are identified, researchers and practitioners should also consider how to implement them county-wide or community-wide to minimize resources needed. A multidisciplinary team is needed to plan and coordinate an initiative that incorporates promoting ATS initiatives, gaining buy-in, physically changing the surrounding environment, and creating policies.

## Figures and Tables

**Table 1:** Characteristics of Schools and Communities and of Active-Transport-to-School Initiatives at Two Elementary Schools, North Carolina, 2005

Characteristic	School A	School B
**Location**	Small town, northeastern North Carolina	Central area of a city, central North Carolina
**Population of census block group^a^ where school is located**	2396	2882
**Density of school census block group**	1165 persons/square mile	4393 persons/square mile
**Number of students**	319	712
**% Race/ethnicity at school**		
White	73	<1
Black	23	80
Asian	<1	0
Hispanic	2	19
**% Receiving free or reduced-price lunch at school**	70	88
**5 P strategies**		
Preparation	None	Delayed rolling out program for a year to better prepare; attended workshops; did research
Promotion	Initial promotion of active-transport-to-school program through news media and meetings with the parent–teacher association and school principal	Promotion to community and parents through news media during WTS Week, and promotion to students and parents through information about the Feeling Good Mileage incentives program
Program	None	WTS Day; WTS Week (including pedestrian/bike safety workshop); walking incentive program; teacher fitness committee
Policy influence	Reduced school bus transport to create "no transport zones"; established a school zone with a reduced speed limit; provided a crossing guard	Established a school zone with a reduced speed limit; relocated a crossing guard; established "no transport zones" for school buses
Physical project	Added sidewalk infrastructure	Added sidewalk infrastructure; installed additional safety signals; removed barriers to sidewalks

WTS indicates Walk to School; 5 P strategies, the Active Living by Design Community Action Model strategies framework.

a Census block group is the smallest geographical unit for which the U.S. Census Bureau publishes sample data.

**Table 2:** Job Title and Location of Key Informants Interviewed on Active-Transport-to-School Initiatives at Two Elementary Schools, North Carolina, 2005

Key Informant	School A	School B
School system official	1	1
School principal	2	1
School administrator	0	1
Physical education teacher	1	1
Parent representative	1	0^a^
City official or council member	1	1
Safety official	1	1
City planner	1	0
City transportation planner	0	1
State school transportation planner	(Same person knowledgeable about both schools' active-transport-to-school initiatives)	
Total	9	8

a Two of the key informants interviewed for School B had children or grandchildren who attended the school either currently or in the past.

**Table 3:** Selected Comments on Active-Transport-to-School Initiatives at Two Elementary Schools, by the Strategies of the Active Living by Design Community Action Model Framework, North Carolina, 2005

Strategy/School	Comment
**Preparation**	
School A	"[The parents] got used to the buses and they liked the buses, so when the sidewalks came they weren't thrilled. So I don't know what else could've been done that, you know, maybe some type of education on benefits of the sidewalk. Other than they're there, so you need to use them, 'cause we're not gonna put your child on the bus."
	"[ATS] wasn't promoted by the school because at the time we had some parents who were very upset that their children had been riding a bus for a year and a half and now all of a sudden they were told they can't ride the bus."
School B	"So you have to attend the workshops, really understand what's going on and then start making [plans] — well this [is] what I think we should do. Because see last year . . . we just weren't ready yet, so we did more research and got ourselves ready. And that's why this year's our year to really kick it off."
**Promotion**	
School A	"All the focus is on the cars, and it seems to be an afterthought that the walking's mentioned, or it doesn't seem to get the billing that it should."
	"It's educating the parents to get them to agree to it, and a lot of times educating the parents is a whole lot more difficult than educating children."
	"I think we talked about walking and biking safety to and from school, but as far as [efforts] being done to promote it, that was more of a parental decision, whether or not they wanted their children to be walking on the sidewalks to school or not."
	"The teachers and the administrators have got to believe in what they're selling."
School B	"Just making walking to school more attractive. That's the whole, that's one of the major things we want to do, just make it more attractive."
	"Our principal has been really supportive of it, and I think when your leader kind of sets the tone, it kind of [trickles] down with the staff members, [gets] some community members involved and [gets] the students and teachers excited about it."
	"[The students] go home, talk to their parents about it, 'cause, you know, they have the greatest influence on their parents."
**Programs**	
School B	"I think another [big issue] would be how well the school itself handles the program. Do they do the groundwork that's necessary to organize it and make it so it's easy for parents to do or — and easy for the kids to do?"
**Policy influence**	
School A	"When I'd come up [the] road in the morning, I'd see some parents walk out to the sidewalk and they could watch their child walking down the sidewalk up to school. And then, they could safely cross with the crossing guard."
School B	"You know, with any initiative, especially in schools, the first thing — well how much more work is involved in it? . . . Is it gonna cost the school any more money?"
**Physical projects**	
School A	"There is an area back over on the other side of [the street] and those children are not a mile and a half away from school, but there's no sidewalk and no way for them to safely cross [the street], so they are picked up by bus."
School B	"There needs to be sidewalks to walk on because no parent in their right mind is gonna send their kid down a drainage ditch to school."

ATS indicates active transport to school.
